# Response to “Comment on Prevalence and Influencing Factors of Malnutrition in Diabetic Patients: A Systematic Review and Meta‐Analysis”

**DOI:** 10.1111/1753-0407.70066

**Published:** 2025-03-03

**Authors:** Tong Zhang, Yuxia Ma, Lin Han

**Affiliations:** ^1^ Evidence‐Based Nursing, School of Nursing Lanzhou University Lanzhou China; ^2^ Department of Nursing Gansu Provincial Hospital Lanzhou China

Dear Editor,

We thank the authors for their insightful comments and for recognizing that our manuscript provides valuable contributions to the field of clinical nutrition [1].

Firstly, we acknowledge that meta‐regression can provide additional insights into heterogeneity, but its feasibility and reliability in our study were constrained by the inconsistent and limited reporting of key covariates, such as sample characteristics, across the included studies, and current meta‐analysis studies on single‐group rates are all highly heterogeneous [2, 3]. Additionally, even meta‐regression analysis cannot completely resolve heterogeneity, which is inherent to meta‐analysis investigating prevalence rates. Heterogeneity is now widely recognized and accepted as a standard challenge in such studies and is one of the issues to be addressed by future methodologists [4].

Secondly, using Egger's test and funnel plots to assess publication bias are widely adopted methods, and while tools such as Doi plots and the LFK index may provide alternative methods for detecting publication bias, these methods have not been universally used in meta‐analyses. We acknowledge that prediction intervals (PIs) can convey the range of effects expected in future studies, but calculating and interpreting PIs relies on normality assumptions, which may be difficult to guarantee. Importantly, retaining our original analysis methods does not alter the conclusions of this paper, which is why we opted to maintain them.

Thirdly, regarding malnutrition assessment tools, we note that a meta‐analysis of 83 studies identified more than 30 nutritional assessment tools, none of which are universally applicable or specifically developed for diabetic patients [5]. We recognize that pooling results from diverse tools introduces significant heterogeneity, but limiting analysis to stratified results would constrain the exploration of factors influencing malnutrition in diabetic patients. To address this, we performed subgroup analysis based on assessment tools. Furthermore, we also advocate for the development of a standardized malnutrition assessment tool tailored for diabetic patients to enhance consistency and comparability across studies.

Finally, we agree that the analysis of some influencing factors, such as smoking, education level, and diabetic foot infection, was limited by small sample sizes. Future studies should focus more on the impact of these factors on the nutritional status of diabetic patients. Additionally, future analysis should aim to incorporate confounding variables, including socioeconomic status, dietary patterns, and psychological factors, to provide a more comprehensive understanding of malnutrition risk.

In conclusion, we thank the authors for their comments on the manuscript and for providing valuable insights. We hope these clarifications address the issues raised and further illuminate our analytical approach and findings.

## Conflicts of Interest

The authors declare no conflicts of interest.
